# Microstructure and Erosion Wear of In Situ TiC-Reinforced Co-Cr-W-C (Stellite 6) Laser-Cladded Coatings

**DOI:** 10.3390/ma17133101

**Published:** 2024-06-25

**Authors:** Jacek Górka, Tomasz Poloczek, Damian Janicki, Aleksandra Lont, Sławomir Topór, Marcin Żuk, Agnieszka Rzeźnikiewicz

**Affiliations:** Welding Department, Faculty of Mechanical Engineering, Silesian University of Technology, Konarskiego Street 18A, 44-100 Gliwice, Poland; damian.janicki@polsl.pl (D.J.); aleksandra.lont@polsl.pl (A.L.); slawomir.topor@polsl.pl (S.T.); marcin.zuk@polsl.pl (M.Ż.); agnieszka.rzeznikiewicz@polsl.pl (A.R.)

**Keywords:** Stellite 6, in situ, laser cladding, erosive wear, TiC, cobalt-based alloys

## Abstract

The article presents research results on the possibility of shaping the structure and properties of Co-Cr-W-C-Ti alloys (type Stellite 6) using laser cladding technology. Cobalt-based alloys are used in several industries because they are characterized by high erosion, abrasion, and corrosion resistance, retaining these properties at high temperatures. To further increase erosion resistance, it seems appropriate to reinforce material by in situ synthesis of hard phases. Among the transition metal carbides (TMCs), titanium carbide is one of the hardest and can have a positive effect on the extension of the lifetime of components made from cobalt-based alloys. In this article, concentration of C, W, and Ti due to the possibility of in situ synthesis of titanium carbides was subjected to detailed analysis. The provided research includes macrostructure and microstructure analysis, X-ray diffraction (XRD), microhardness, and penetrant tests. It was found that the optimal concentrations of Ti and C in the Co-Cr-W-C alloy allow the formation of titanium carbides, which significantly improves erosion resistance for low impact angles. Depending on the concentrations of titanium, carbon, and tungsten in the molten metal pool, it is possible to shape the alloy structure by influencing to morphology and size of the reinforcing phase in the form of the complex carbide (Ti,W)C.

## 1. Introduction

Cobalt alloys are attracting constantly growing interest as materials used in many industries due to high resistance to abrasives, erosive wear, and corrosion in aggressive environments, combined with the ability to work in high temperatures (up to 1000 °C). One of the most popular varieties of cobalt alloy is the Co-Cr-W-C alloy, which is available in the industry under many trade names (Stellite 6, MetcoClad 6). It is most often used as a material to produce coatings, where heat sources such as a gas flame, electric arc, plasma, or laser beam can be used [1,2,3,4]. Particular attention should be paid to the laser cladding process, which is characterized by unique properties, such as the possibility of precise control of generated heat, the ability to limit thermal deformations, and short crystallization time enabling obtaining fine-grained structures or metastable phases. The described technology, despite the relatively high costs of the device, has several advantages, which is why there has been a constant increase in interest in technological processes related to cladding [5,6,7,8]. One of the alternatives to laser cladding is electrospark deposition (ESD) technology. ESD offers high precision, low thermal input, minimal dilution, low device cost, and versatility across various materials. However, it has a slower deposition rate, often requires post-processing for a smooth surface finish, and is limited in coating thickness. In contrast, laser cladding provides a higher deposition rate, excellent control over deposition parameters, a smoother surface finish, and the possibility of processing a wide range of materials [9,10].

The Co-Cr-W-C alloy owes its useful properties to its structure, which consists of the γ-Co phase and eutectics containing M_7_C_3_ and M_23_C_6_ carbides. However, due to the high cost of material and constantly increasing production costs, there is interest in further improving their wear resistance [11,12,13]. A particularly interesting approach to increase the erosion resistance and tribological resistance of Co-Cr-W-C alloy coatings is the in situ synthesis of carbide phases by adding into the base powder elements with a strong carbide-forming tendency, which lead to metallurgical reactions in the molten metal pool, enabling the formation of reinforcing phases during the crystallization process. The main advantages of in situ synthesis of reinforcing phases compared to the ex situ method are unoxidized and uncontaminated interfacial surfaces between the matrix and reinforcing phase, ensuring high-quality coatings. Additionally, in situ phases in metal matrix composites (MMC) are characterized by high thermodynamic stability in the alloy matrix, showing better properties during operation at elevated temperatures [14,15,16,17,18].

Research reported by Wang et al. [11] on Stellite 6 alloy reinforced with 27.13% TiFe + 17.86% Cr_3_C_2_ showed a fivefold increase in wear resistance compared to the pure Stellite 6 alloy due to the formation of a TiC phase, which also contributed to an increase in average hardness up to 658 HV. An experiment conducted by Acevedo et al. [19] on Stellite 6 coatings reinforced by nano-TiC particles showed that the addition of 2% TiC content resulted in a significant improvement in wear resistance. Shahroozi et al. [20] concentrated on tungsten inert arc welding (TIG) of Stellite 6 alloy with 10–40 wt% of TiC. The results of the investigation showed that the addition of TiC improved the alloy coatings’ hardness and wear resistance with an increase in TiC concentration. In the study conducted by Wu et al. [21] on Stellite 6 modified by Ti + Ni, the morphology of the carbides was altered by the addition of titanium, changing from M_23_C_6_ eutectic carbides to isolated TiC. Furthermore, the chromium, which was initially present in eutectic carbides, segregates in the cobalt matrix as a result of titanium’s reaction with carbon, lowering the stacking-fault energy (SFE) and causing the formation of the ε-Co phase, while the addition of nickel stabilizes the γ-Co phase by increasing the SFE.

The relationship between erosion resistance and the chemical composition of Stellite alloys has not yet been systematized or published in a broader publication. Research conducted by a group of scientists represented by Levin et al. [22] concerned the erosion of cobalt superalloy clads (focusing on the Stellite 6 and Ultimet from the group of cobalt alloys). It has been stated that under conditions of stable erosive wear at elevated temperatures (400 °C), the Stellite 6 alloy shows reduced resistance to erosive wear compared to materials such as 316L stainless steel, Inconel-625, or Ultimet alloy. The author demonstrated the lack of dependence of erosion loss on the impingement angle, stating that for the Stellite 6 alloy at a temperature of 400 °C, erosion loss occurred as a result of plastic deformation of the material.

Nsoesie et al. [23] showed that Stellite alloys owe their erosion-resistance properties to the high content of carbides, while alloys with reduced carbon content form the intermetallic compounds Co_3_Mo and CoMo_6_, which have the same strengthening mechanism as carbides, ensuring low erosion loss. The analysis of the tests carried out allowed the conclusion that at an erodent impingement angle of 30°, the resistance to erosive wear is closely related to the carbon content and hardness of the material. This is due to the increase in resistance to microcutting processes as the surface hardness increases. Despite the high content of relatively large-sized (W,Co)C carbides in the microstructure, the samples were characterized by reduced erosion resistance compared to materials containing fine Cr_7_C_3_ carbide precipitates in their microstructure. This is due to the fact that under the constant impact of the erodent, large carbides are more easily cracked and torn out of the matrix due to their high brittleness. This is especially evident in the case of large (W,Co)C precipitates.

The presented literature review indicates that the in situ synthesis of in situ titanium carbides in cobalt alloys can contribute to a significant increase in resistance to erosive wear [24,25]. In this study, the results regarding the possibility of shaping the structure and properties of cobalt-based alloys (type Stellite 6) fabricated by laser cladding were provided to enhance the erosion resistance wear of obtained coatings. The effect of the concentration of elements in the Co-Cr-W-C-Ti alloy on the possibility of in situ synthesis of titanium carbide was subjected to a detailed analysis. In particular, the effect of the addition of titanium and carbon on the ability to control the reinforcing-phase content and morphology was investigated. Subsequently, the produced coatings were subjected to erosion tests and determination of the erosion wear mechanism.

## 2. Materials and Methods

### 2.1. Materials and Laser Processing

Laser cladding of the Co-Cr-W-C alloy was performed on 10 mm thickness S355JR steel (Cognor, Stalowa Wola, Poland), meeting the requirements of EN 10025-2 standard [26]. Samples were subjected to grinding and degreased using ethyl alcohol (Stanlab, Lublin, Poland). For the cladding process, powders of Stellite 6 (Metcoclad 6 gas atomized powder, Oerlikon, Westbury, NY, USA), 99.0% Ti (Amperit 154, H.C. Starck, Germany), and 99.5% C (1.04206.9025, Merck, Germany) were used. Before the cladding process, the powders were mixed in a vertical planetary ball mill for 30 min and dried for 1 h at temperature of 50 °C. The fraction of powder particles was in the range of 45–106 µm (according to ASTM B214-22 standard [27]). The chemical compositions of the powder mixtures are presented in Table 1.

**Figure 1:**
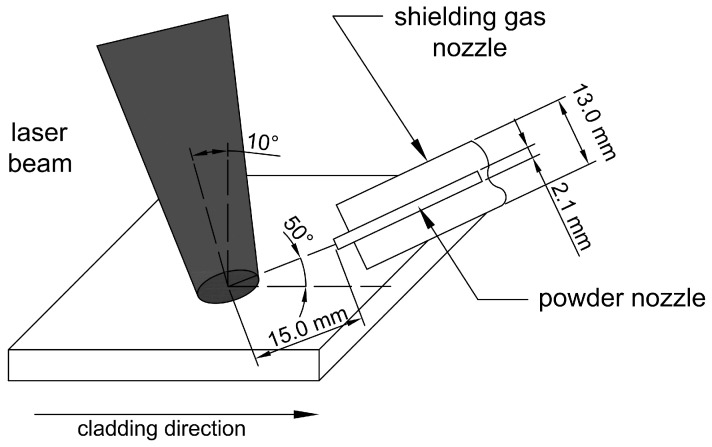
Scheme of laser cladding process.

The powder laser cladding process was carried out on a test stand equipped with a solid-state laser (TRUMPF, Ditzingen, Germany) with a numerically controlled laser head positioning system. The laser beam was focused 30 mm above the surface of the workpiece material to avoid excessive penetration of the base material and to increase the surface heating area. This value was selected based on preliminary geometric measurements of the cross-section of the obtained clads. The laser head was tilted 10° from the vertical position, and the powder was injected into the molten pool through a 2.1 mm diameter cylindrical nozzle from 15 mm. As a shielding and transporting gas, argon with a purity of 99.999% was used (flow rates—15 L/min and 3 L/min, respectively). Samples were not preheated before the cladding process, and the interpass temperature was less than 50 °C. Scheme of the laser cladding process is shown in Figure 1. Based on preliminary tests, the optimal cladding parameters were determined to be as follows: laser power of 1750 W, cladding speed of 200 mm/min, and powder feed rate of 35 mg/min. The linear energy, calculated as the laser power divided by the cladding speed, was 525 J/mm.

### 2.2. Non-Destructive Tests and Macroscopic Examination

Penetrant tests were carried out in the color contrast technique with 10 min dwell and 15 min development time. For this test, the cleaner MR 79, penetrant 68 NF, and developer MR70 (MR Chemie, Unna, Germany) were used. Samples’ temperature during penetrant examination was 23 °C, while the light intensity was 920 lux. Macroscopic observations were performed on a stereoscopic optical light microscope SZX9 (Olypmus, Tokyo, Japan). Based on the macro photographs, the geometric parameters of the coatings were determined using AutoCad 2024 software (Autodesk, CA, USA). The dilution of coatings was determined using Equation (1), where *F_BM_* is the cross-sectional area of the melted substrate and F_c_ is cross-sectional area of the coating.

(1) $$U=\frac{F_{BM}}{F_{BM}+F_{C}}\times 100\;[\%]$$

Microscopic observations were performed on a Phenom World Pro scanning electron microscope (SEM) equipped with energy-dispersive spectroscopy (EDS) for chemical composition analysis. The surface area of individual phases was calculated using the planimetric method in 6 areas for each of the samples considering areas with secondary thermal cycles due to the 50% overlap between each of the passes. Transmission electron microscopy (TEM) examinations were performed on Titan 80/300 (FEI, Hillsboro, OR, USA), using high-resolution transmission electron microscopy (HRTEM) and scanning transmission electron microscopy (STEM). To determine the obtained phases, diffraction images taken from selected areas of the sample using the selected-area electron diffraction (SAED) technique were obtained. Thin foil for TEM investigation was prepared using the Xe-PFIB (plasma focused ion beam) technology. The X-ray diffraction (XRD) analysis was performed using a PANalytical X’Pert PRO MPD diffractometer (Malvern Panalitycal, Malvern, UK) with a cobalt anode. The diffraction patterns were obtained in continuous scan mode (2ϴ range of 25° to 130°) with a step size of 0.1444° and 0.026 s counting time per step.

**Figure 2:**
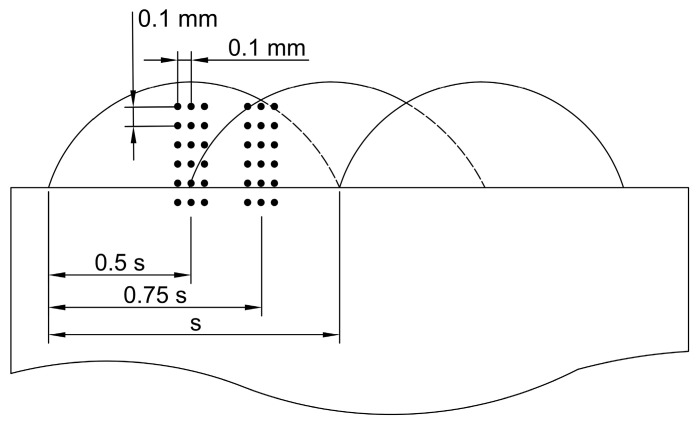
Schematic diagram of microhardness measurement lines.

Microhardness tests were carried out using the Vickers method on cross-sections of coatings. For this purpose, a Wilson Wolpert 401 MVD (Wilson Instruments, Instron Company, Norwood, MA, USA) hardness tester was used. The measurements were carried out with a load of 300 g and a dwell time of 10 s. The tests were conducted across six measurement lines (3 in the laser pass axis with a distance between the lines of 0.1 mm and 3 in the interpass area with a distance between the lines of 0.1 mm), as shown in Figure 2.

Erosion resistance tests were performed on a testing stand meeting the requirements of ASTM G76-04 [28] standard. As an erodent, Al_2_O_3_ powder with a grain size of 50 µm was used. The particles were transported in a stream of dry air with an abrasive particle velocity of 30 m/s, while the powder flow rate was 2 g/min. The erodent distributing nozzle was located 10 mm above the test surface with a test duration time 10 min for each sample. Erosion resistance examinations were carried out for two impingement angles—30° and 90°. Mass loss calculations were carried out on a WAX 60/220 laboratory scale (Radwag, Radom, Poland). Parameters of erosion rate and erosion value were calculated based on Equations (2) and (3).

(2) $$Erosion\;rate=\frac{mass\;loss\;[g]}{test\;time\;[min]}$$

(3) $$Erosion\;value=\frac{volume\;loss\;[{mm}^{3}]}{total\;mass\;of\;abrasive\;[g]}$$

## 3. Results and Discussion

### 3.1. Penetrant Tests

**Figure 3:**
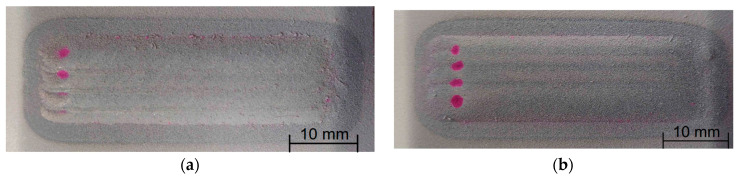
Surfaces of the samples after penetrant test: (**a**) M6; (**b**) M6TiC; designation in accordance with Table 1.

The penetrant tests, presented in Figure 3, showed only false indications for all of the samples at the end of the cladding process. This is the result of craters in these areas and the powder particles that have not been completely melted, making it impossible to clean the excess of penetrant from these areas. The lack of linear indications shows the high quality of the coating M6TiC, where the addition of carbon and titanium did not weaken the interface bonding between matrix and reinforcing phases, which is very often observed in titanium carbide-reinforced MMC coatings [12].

### 3.2. Macrostructure

**Figure 4:**
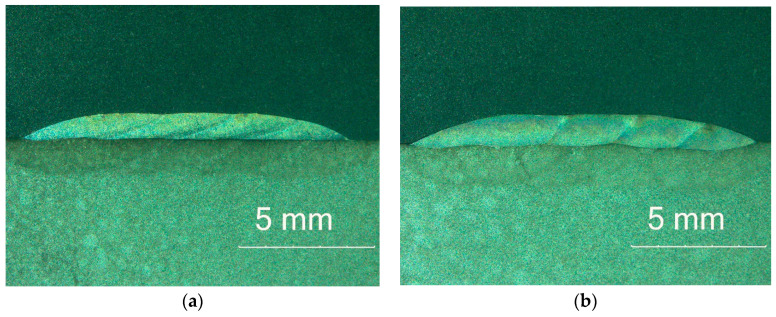
Coatings’ macrostructure on cross-section: (**a**) M6; (**b**) M6TiC; designation in accordance with Table 1.

Regardless of the different chemical compositions of coatings, samples were characterized on cross-section by the absence of any welding imperfections in the form of cracks, which are often observed in MMC coatings with carbide phases in their structure, as presented in Figure 4. The selected 50% overlap between each of the passes resulted in very low surface waviness, which is an important aspect due to the possibility of reducing potential machining. Macrostructure analysis showed that the depth of fusion into the base material was characterized by the highest value for the first pass for each coating. This is a result of cladding technology, where, during the first pass, the substrate material undergoes intense melting, while the overlapping of each subsequent pass causes partial melting of the previously deposited layers. The parameters of width, height, and cross-sectional area of the coatings increased with the addition of graphite, as presented in Table 2. This can be attributed to the fact that carbon increases the absorption of laser radiation [29], which leads to an increase in the surface area of the molten pool.

### 3.3. Microstructure

**Figure 5:**
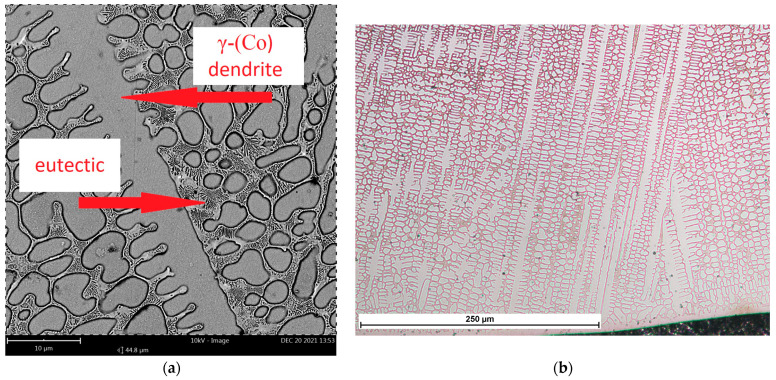
M6 coating microstructure: (**a**) phase composition; (**b**) dendrite growth from fusion line.

**Figure 6:**
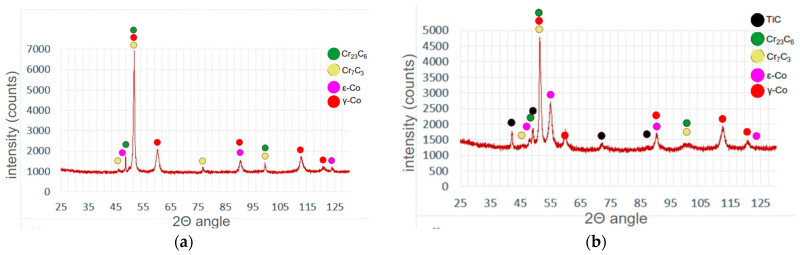
XRD results: (**a**) M6; (**b**) M6TiC; designation in accordance with Table 1.

A microstructure analysis of M6 coating (made of a commercial hypoeutectic Co-Cr-W-C alloy—type Stellite 6) was performed for comparative analysis of changes in the microstructure under the influence of Ti and C alloying elements in sample M6TiC. Microstructural analysis of M6 coating shows that in the first stage, the alloy crystallizes as dendrites of the solid solution of the γ-(Co) phase (FCC) in Figure 5a. In the next stage, the eutectic crystallizes in the interdendritic spaces, consisting of M_7_C_3_/M_23_C_6_ carbide precipitates and the γ-(Co) phase. XRD studies, presented in Figure 6a, indicate that further cooling causes a partial transformation of γ-(Co) to the ε-(Co) phase (HCP). The results are consistent with published data on the Co-Cr-W-C alloy cladding process, where Jackson [30] revealed in quantitative XRD analysis 2–3% of the ε-(Co) phase in coatings. At the fusion line, columnar dendrites with long lengths up to 300 μm can be observed (Figure 5b), which is typical for low undercooling [31]. The direction of their growth is directly related to the heat flow direction during the crystallization process, where the growth of dendrites starts from the fusion line and extends towards the surface of the coating. Typical columnar dendrites were not observed in the near-surface zone, which is directly related to the change of heat flow direction in these regions during cooling.

**Figure 7:**
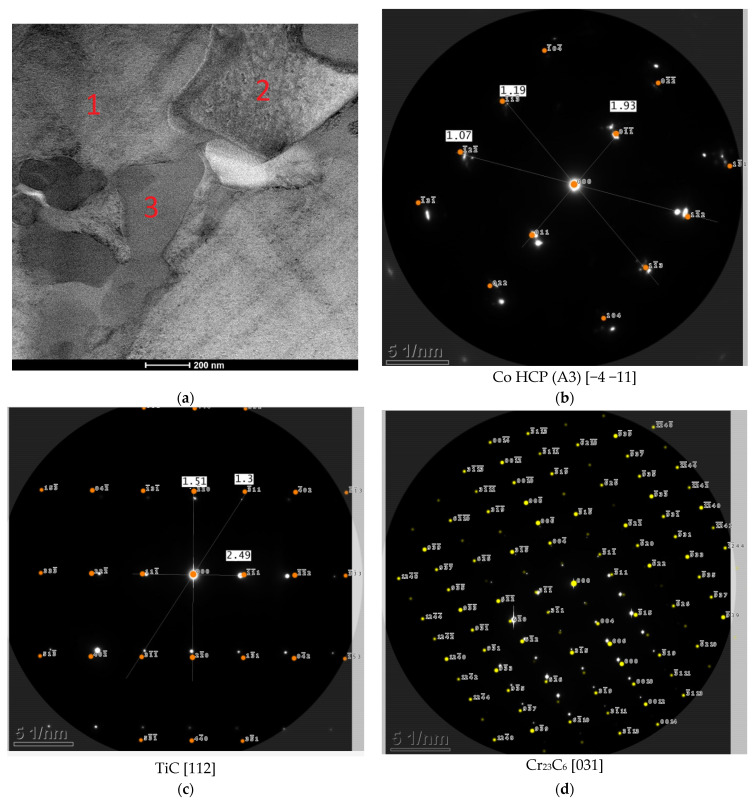
TEM image of M6TiC coating: (**a**) STEM HAADF image; (**b**) SAED diffraction pattern from point 1; (**c**) SAED diffraction pattern from point 2; (**d**) SAED diffraction pattern from point 3.

**Figure 8:**
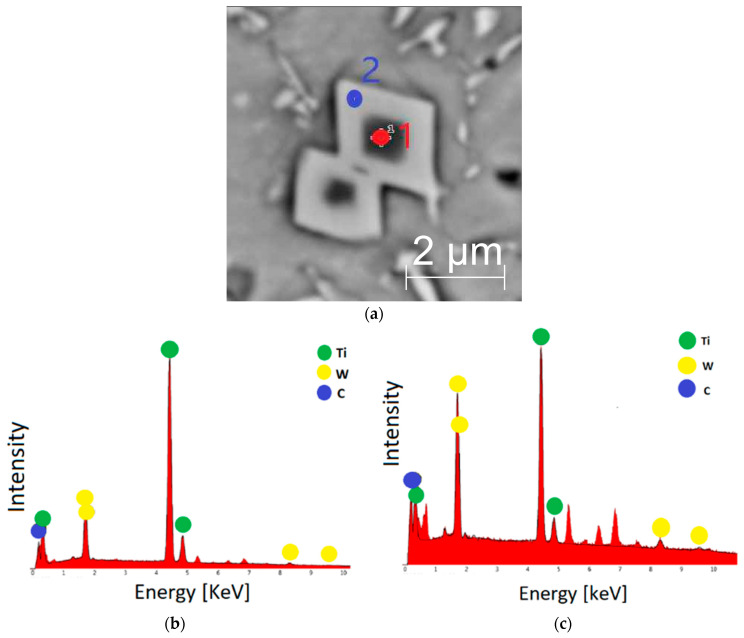
M6TiC coating: (**a**) typical morphology of primary (Ti,W)C carbide; (**b**) EDS spectrum from point 1; (**c**) EDS spectrum from point 2.

The addition of titanium and carbon led to significant changes in the microstructure of the M6TiC coating by crystallization of the reinforcing phase in the form of primary titanium carbides. Titanium, which possesses a high affinity for carbon, enabled the synthesis of TiC carbide directly in a molten metal pool, which was in agreement with the presented calculations of the phase equilibrium system for cobalt alloys by Bandyopadhyay et al. [32]. The carbon concentration of 2.1 wt% and titanium concentration of 4.0 wt% ensured the crystallization of titanium carbide as the primary phase, as evidenced by the cubic morphology of the observed precipitates and their presence within the primary dendrites of the γ-(Co) phase, rather than in the eutectic regions, as presented in Figure 7a. The analysis of the reinforcing primary carbide phase revealed that tungsten atoms dissolve in the carbide’s crystal lattice, as confirmed by microstructure images (Figure 8a). The SEM EDS analysis of the coating (Figure 8b,c) indicates that primary carbides initially nucleate at lower tungsten concentrations. However, as they grow, an increasing number of tungsten atoms dissolve into the titanium carbide crystal lattice, resulting in gradient concentrations of Ti and W in carbide.

**Figure 9:**
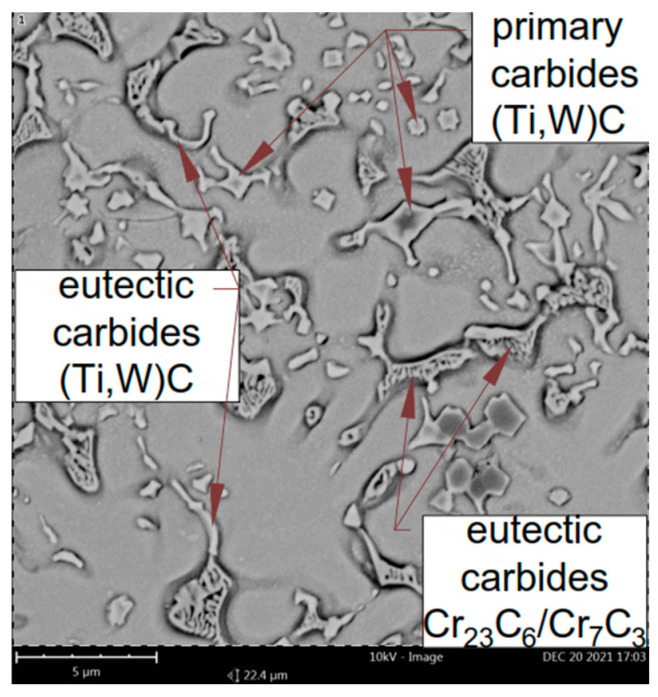
Microstructure of M6TiC coating.

After the crystallization of primary carbides, the eutectic consisting of (Ti,W)C carbide and dendrites of the γ-(Co) phase crystallize; then, they partially transform into the ε-(Co) phase, as shown in XRD analysis (Figure 6b) and TEM investigations (Figure 7b). The nucleation of eutectic (Ti,W)C carbide begins from the primary carbides. They exhibit different morphology, as laths approximately 1–2 μm in length, as presented in Figure 9. The average volume fraction of eutectic (Ti,W)C phase precipitates in the coating was about 6.5%, which is a similar value to the primary carbides: 5.9% (Table 3). With the addition of titanium in M6TiC coating, the quantity of eutectic chromium carbide precipitates significantly decreases to a 10.1% volume fraction. This observed relationship results from the fact that titanium has a higher affinity for carbon than chromium, leading to a reduction in the volume fraction of eutectic chromium carbides. Consequently, the solid solution matrix is characterized by a higher chromium concentration, which promotes the transformation of γ-(Co) → ε-(Co) in the cobalt solid solution. 

In the analyzed M6TiC coating, the typical columnar growth of dendrites from the fusion line (visible in sample M6) was inhibited. This is due to the high thermodynamic stability of (Ti,W)C carbides, which precipitate in the first stage of the crystallization process and, as previously mentioned, serve as a substrate for nucleation and growth of dendritic γ-(Co) solid solution crystals.

### 3.4. Microhardness

**Figure 10:**
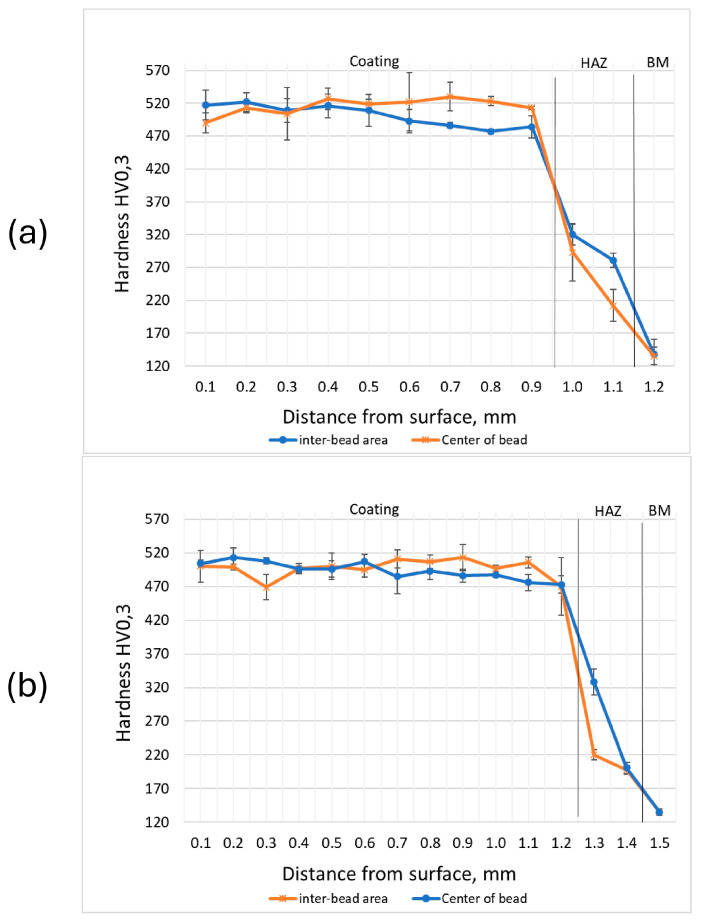
Microhardness on cross-section: (**a**) sample M6; (**b**) sample M6TiC; designation in accordance with Table 1 (HAZ—heat-affected zone; BM—base material).

The microhardness distributions on the cross-section of the coatings along the laser pass axis and in the interpass zone are presented in Figure 10. The microhardness analysis of the interpass area of sample M6 revealed a slight decrease in hardness, where, due to the additional thermal cycle, grain growth of the cobalt solid solution and eutectic phases occurs. The hardness of the heat-affected zone for all samples ranged from 190 to 328 HV_0.3_, which then stabilized in the base material at a value of 120 to 140 HV_0.3_. The M6TiC sample exhibited higher uniformity of hardness distribution along the cross-section of the coating. This can be directly attributed to the absence of microstructural changes in the areas affected by the subsequent thermal cycle during multi-pass cladding, where high thermal stability of the (Ti,W)C phases ensured structural homogeneity and, consequently, stability of the hardness parameter distribution. 

The presented data show slight changes in the microhardness parameter with variations in the chemical composition of the powders used for the laser cladding process (samples M6 and M6TiC). The microstructure of the M6 coating consisted of dendrites of the cobalt solid solution and a eutectic containing carbides of the Cr_7_C_3_ and Cr_23_C_6_ types, whose uniform distribution within the alloy structure directly contributed to the high hardness of the coating, ranging from 469 to 530 HV_0.3_, which is consistent with the research of Kołodziejczak et al. [33]. In the case of the M6TiC sample, the high hardness was due to the presence of both chromium carbide eutectics and titanium carbides in the structure.

### 3.5. Solid Particle Erosion Tests

Erosion resistance tests have shown that addition of Ti and C to the Co-Cr-W-C alloy significantly influences erosive wear. The in situ synthesis of (Ti,W)C carbides enhanced the erosion resistance compared to the base Co-Cr-W-C alloy (M6 sample) at an impingement angle of 30°, while maintaining high erosion resistance at a 90° angle, Table 4.

**Figure 11:**
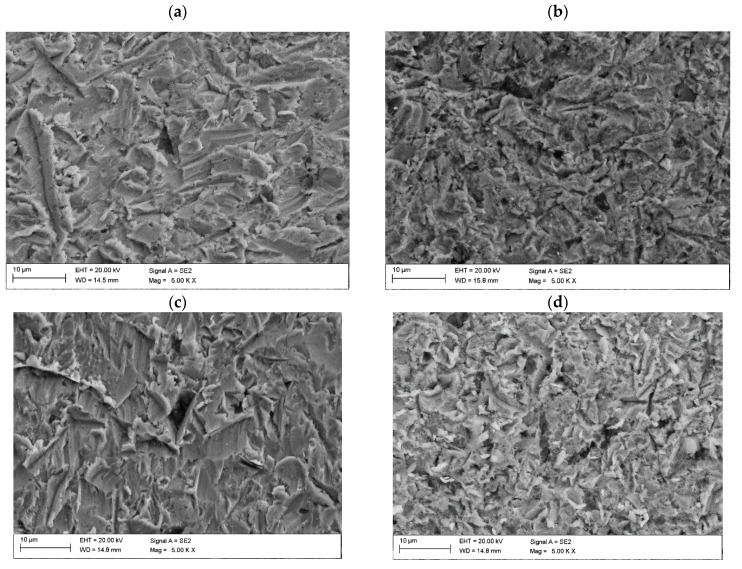

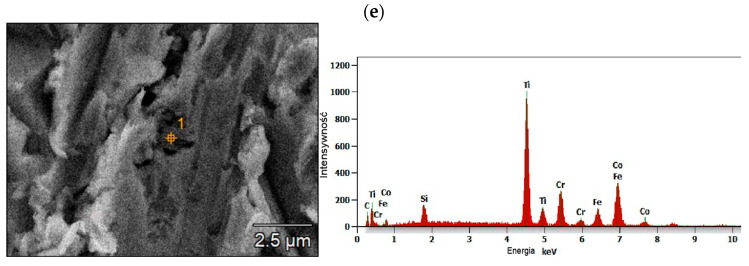
SEM image after erosion tests: (**a**) sample M6 30° impingement angle; (**b**) sample M6 90° impingement angle; (**c**) sample M6TiC 30° impingement angle; (**d**) sample M6TiC 90° impingement angle; (**e**) sample M6TiC showing chipped TiC carbide with an EDS diffractogram of point 1.

Solid particle erosion tests conducted at an erodent impingement angle of 90° revealed a similar erosion rate for all of coatings, with an average value of 25 mg/min. For the same impingement angle, the erosion value for sample M6 was 0.015 mm^3^/g, while the M6TiC sample exhibited a value of 0.016 mm^3^/g. Considering the standard deviation, it can be concluded that the described samples exhibited the same level of erosion value. For the 30° erodent impingement angle, significant changes in the erosion value could be observed. In comparison to the base coating M6, the M6TiC sample exhibited a significant decrease in erosion value by 35% (0.012 mm^3^/g), Table 4. The conducted studies did not reveal a clear trend between the hardness of the coatings and their resistance to erosion wear. The presence of the titanium carbides and chromium eutectic carbides in the structure of sample M6TiC did not significantly affect the erosion value at an erodent impingement angle of 90° but markedly reduced the intensity of erosion at a 30° impingement angle. The analyzed composite coatings, due to their complex microstructure consisting of a matrix and reinforcing phase in the form of titanium carbides, exhibited two distinct erosion wear mechanisms. The first mechanism involved plastic deformation of the matrix material (visible grooves and fragments of plastically deformed material), indicating an erosion wear mechanism typical for ductile materials (Figure 11a,b). The second mechanism involved cracking of the reinforcing phase, characterized by high hardness, which is a typical erosion wear mechanism observed for brittle materials (Figure 11c–e). SEM imaging of the M6 sample after erosion tests, as presented in Figure 11a, shows grooves caused by microcutting by erodent particles, as a result of material plastic deformation. This observation is consistent with the research of Sidhu et al. on the solid particle erosion of Stellite 6 [34]. Changing the erodent impingement angle to 90° did not alter the observed surface damage; however, the visible grooves were significantly shorter in length, as presented in Figure 11b. In the M6TiC coating containing the reinforcing phase in the form of (Ti,W)C carbides, cracking and spalling of the hard reinforcing phase particles was observed, combined with microcutting of the matrix material (Figure 11e).

## 4. Conclusions

Based on the conducted studies on the possibility of in situ synthesis of TiC in Co-Cr-W-C alloys (type Stellite 6), the following conclusions can be formulated:

- The laser cladding process using Co-Cr-W-C powder (type Stellite 6) with addition of Ti and C makes it possible to obtain homogenous coatings with composite structure reinforced by an in situ TiC phase.
- The addition of titanium to the Co-Cr-W-C alloy leads to the crystallization of primary (Ti,W)C carbides, characterized by a gradient tungsten concentration, where the tungsten content increases with the growth of the carbide.
- The addition of titanium to the Co-Cr-W-C alloy leads to the reduction of eutectics composed of chromium carbides. This phenomenon occurs because titanium has a greater affinity for carbon than chromium, resulting in the precipitation of primary (Ti,W)C carbides in the first stage of the crystallization process.
- The presence of the reinforcing phase in the form of (Ti,W)C carbides results in increased erosion resistance at an erodent impingement angle of 30° (a 35% decrease in the erosion value parameter compared to the base coating made of Co-Cr-W-C alloy) while maintaining high erosion wear resistance at an erodent impingement angle of 90°.
- The composite coating reinforced by in situ titanium carbide exhibits two distinct erosion wear mechanisms: one typical of ductile materials, resulting in the loss of matrix material, and another typical of brittle materials, where the hard particles of the reinforcing phase undergo cracking.

## Figures and Tables

**Table 1:** Chemical compositions of powder mixtures.

Sample Designation	C	Co	Cr	Fe	Mn	Mo	Ni	Si	W	Ti
M6	1.1	balance	27.3	0.1	0.1	0.1	0.8	1.6	4.4	-
M6TiC	2.1	balance	25.9	0.1	0.1	0.1	0.8	1.5	4.2	4.0

**Table 2:** Geometrical parameters of coatings.

Sample Designation	Width (mm)	Height (mm)	Cross-Sectional Area (mm^2^)	Dilution (%)
M6	12.0 ± 0.1	0.94 ± 0.05	9.27 ± 0.17	1.6
M6TiC	12.7 ± 0.2	1.06 ± 0.05	12.08 ± 0.26	9.6

**Table 3:** Average volume fraction.

Sample Designation (acc. to Table 1)	Average Volume Fraction of Primary (Ti,W)C Carbides [%]	Average Volume Fraction of Eutectic (Ti,W)C Carbides [%]	Average Volume Fraction of Co-Based Solid Solution [%]	Average Volume Fraction of Eutectic Consisting of M_7_C_3_/M_23_C_6_ Carbides [%]
M6	-	-	65.3 ± 0.7	34.7 ± 0.7
M6TiC	5.9 ± 0.7	6.5 ± 0.6	77.5 ± 0.3	10.1 ± 1.1

**Table 4:** Solid particle erosion test results.

Sample Designation (acc. to Table 1)	Average Erosion Rate [mg/min]		Average Erosion Value [mm^3^/g]	
	30°	90°	30°	90°
M6	0.020 ± 0.001	0.015 ± 0.001	0.34 ± 0.02	0.25 ± 0.02
M6TiC	0.014 ± 0.002	0.016 ± 0.002	0.22 ± 0.04	0.25 ± 0.02

## Data Availability

The original contributions presented in the study are included in the article, further inquiries can be directed to the corresponding author.
